# The site of embolization related to infarct size, oedema and clinical outcome in a rat stroke model - further translational stroke research

**DOI:** 10.1186/2040-7378-2-17

**Published:** 2010-09-17

**Authors:** Karsten Overgaard, Rune S Rasmussen, Flemming F Johansen

**Affiliations:** 1 Stroke Unit, University Hospital of Copenhagen, Gentofte Hospital, Hellerup, Denmark; 2 Copenhagen Experimental Stroke Unit, Department of Biomedical Sciences, The Panum Institute, University of Copenhagen, Copenhagen, Denmark

## Abstract

**Background and purpose:**

Reliable models are essential for translational stroke research to study the pathophysiology of ischaemic stroke in an effort to find therapies that may ultimately reduce oedema, infarction and mortality in the clinic. The purpose of this study was to investigate the relation between the site of arterial embolization and the subsequent oedema, infarction and clinical outcome in a rat embolic stroke model.

**Methods:**

Thirty-six male Sprague-Dawley rats were thromboembolized into the internal carotid artery. The site of occlusion was demonstrated by arteriography. Following histological preparation and evaluation, the size of the hemispheres and the infarcts were measured by quantitative histology and planimetry. Another parallel stroke model study was subsequently examined to investigate if the conclusions from the first study could be applied to the second study.

**Results:**

The median size of the infarct was 40% of the ipsilateral hemisphere in both the 19 animals with occlusion localised to the intracranial part of the internal carotid artery and in the 11 animals where the main trunk of the middle cerebral artery was occluded. In 5 animals, occlusion of the extracranial part of the internal carotid artery resulted in significantly smaller infarcts compared to other groups (p < 0.01). Another independent study re-confirmed these results. Furthermore, significant correlations (R > 0.76, p < 0.0001) were found between 1) cortical, subcortical, and total infarct volumes, 2) oedema in percent of the left hemisphere, 3) clinical score before termination and 4) postoperative weight loss.

**Conclusions:**

Distal occlusions of the intracranial part of the internal carotid or middle cerebral arteries resulted in comparable large sized infarctions and oedema. This indicates that investigators do not need a similar number of such occlusions in each experimental group. Contrary to observations in the clinic, distal internal carotid artery occlusions did not result in worse outcome than middle cerebral stem occlusions, but this finding may be explained by the controlled emboli size in this experimental stroke model.

## Introduction

Commonly human stroke patients suffer from direct occlusion of the middle cerebral artery (MCAO), or from occlusions of the internal carotid artery (ICAO), which reduce or prevent sufficient blood flow through the middle cerebral artery (MCA).

Recently, the importance of establishing reliable animal models is clear, as hundreds of effective neuroprotective therapies in animals failed to show beneficial effect in the treatment of human stroke [1]. It has been argued, that the quality of many animal models provided unreliable results due to insufficient study design and type of model, randomisation, blinding, inclusion and exclusion criteria, measurement of physiological parameters etc. [2]. The animal model used in the present investigation is different from several other MCAO animal models by using obstructing autologous blood clots that compromise cerebral arterial circulation in focal brain regions. The advantage of this model in translational stroke research is its similarity to human ischaemic stroke, and this advantage has been exemplified by the fact that this model provided results of thrombolytic efficacy in rats before benefit of human thrombolytic treatment was documented [3-5]. The primary disadvantage of this embolic stroke model is a large variation of infarct volumes, but although the variation in infarct volume makes it necessary to use relatively large animal group sizes, the model may provide a sound basis for translational stroke research.

The main purpose of this study was to investigate the relationship between the site of arterial occlusion and the subsequent size of the resulting infarct in a rat embolic stroke model. If the consequences of embolization in this animal model are shown to be similar to consequences of embolization in humans, the model will be of high translational significance due to its prognostic abilities.

Measurement of infarct volumes generally has been the main endpoint of investigations in animal stroke models, but many factors may interfere with measurements of infarcted tissue such as brain swelling by oedema or brain shrinkage by resorption. In this study we investigated several aspects associated to the embolic location, namely infarct volume, oedema size, mortality and effects of re-embolization. An endpoint to the investigation was to examine whether or not evenly distributed locations of the emboli and the consequently degrees of ischemia among treatment and control groups were necessary to avoid unwanted selection bias, especially when groups are relatively small in numbers. Thus, in the present study, the consequences of emboli localisation especially upon infarct volume were investigated for the first time and further related to both the formation of oedema and the clinical outcome.

## Materials and methods

Animals in this study have previously been investigated for other purposes [6]. Re-using animals to provide extended results and observations is in favour of the methodology encompassed in the concepts of refinement, reduction and replacement within animal laboratory science [7,8]. This ethical methodology corresponds to the aim of medical researchers to use as few animals and as responsibly as possible.

All experiments were approved and conducted according to the guidelines by the Animal Research Committee of University of Copenhagen. Thirty-six male Sprague-Dawley rats weighing from 240 to 420 grams were used. The rats were housed in a 12-hour light-dark cycle at a room temperature of 22 ± 1°C, with unrestricted access to water and chow before and after surgery. Anaesthesia was induced with subcutaneous administration of Hypnorm^® ^(fluanison 4 mg/kg and fentanyl 0.12 mg/kg, Janssen Pharmaceutica, Beerse, Belgium) combined with atropine 0.05 mg/kg and intraperitoneal administration of diazepam 2 mg/kg (Apozepam 5 mg/ml; Apothekernes Laboratorium, Lier, Norway). The body temperature was kept at 37 ± 0.5°C by rectal temperature monitoring and a thermostat controlled heating lamp.

### Surgical procedure and preparation of the emboli

Small white 'arterial-like' elongated autologous microclots rich in fibrin were produced in vitro as reported earlier [4,5,9]. Then by modification and amendment of this method, the suspension of microclots was injected into a 120-cm long PP10 (PP10; ID 0.28 mm, also corresponding to clot diameter) air filled tube [10]. The microclots in the suspension accumulated to several single macroclots of larger and variable length by this method [10]. A macroclot with a length of about 8 mm (0.44 mm^3^) was selected for embolization by cutting a piece of the tube with the macroclot enclosed. Both the right femoral vein and the external carotid artery (ECA) were catheterised, while the pterygopalatine artery was ligated as described earlier [9]. Clotting associated with the inserted catheters was avoided by continuous flow of heparinised (5 IU/ml) saline through the line at the speed of 0.5 ml/h until the catheter was removed at the end of surgery. The mean arterial blood pressure (MABP), blood glucose, haemoglobin, P_a_O_2_, O_2_-saturation, P_a_CO_2 _and pH_a _were measured (Radiometer ABL 2, Copenhagen).

### Embolization produced arterial occlusion documented by arteriography

The right common carotid artery (CCA) was clamped temporarily using a ligature, and the embolus was gently injected through the saline-filled ECA catheter by the use of 0.3 ml saline. This procedure caused embolization of the right hemisphere by occlusion of the internal carotid artery (ICA) or MCA. After this procedure carotid arteriography was performed immediately by bolus injection of 0.20 ml heparinized (5 IU/ml) iohexol (Omnipaque, 300 mg I/ml; Nycomed, Roskilde, Denmark) through the ECA catheter. Angiograms were processed on high-resolution films (Retina XOE; Fotochemische Werke, Berlin, Germany). Since the CCA was unclamped during the angiographic procedure, this procedure did not affect the embolization.

**Figure 1:**
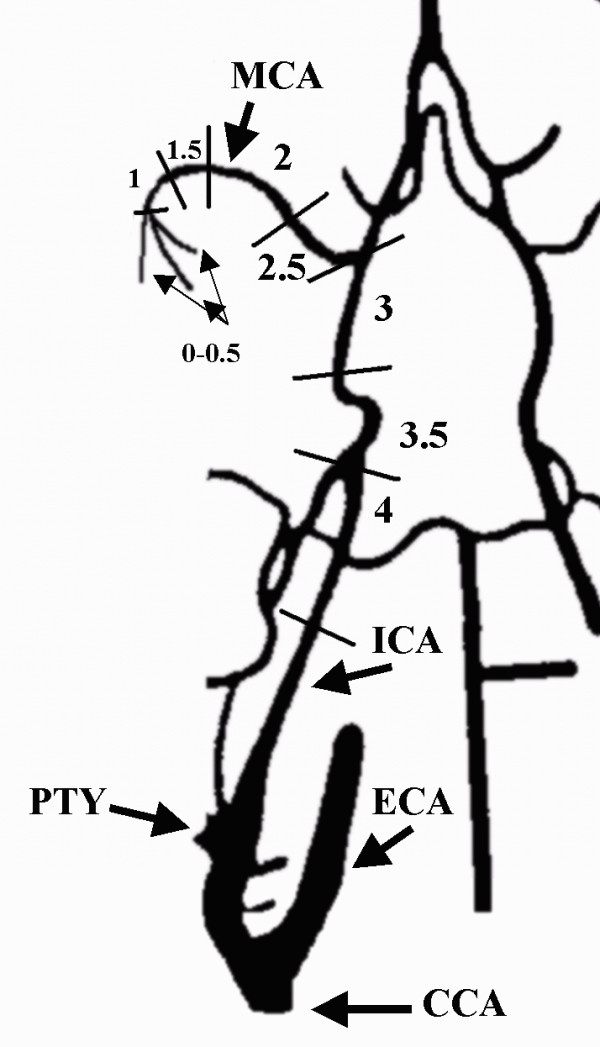
**The ventral aspect of carotid-vertebral arteries in the rat**. All embolized animals should obtain occlusion scores of 1.5 to 4. The following arteries are identified: (clamped) common carotid artery [CCA], (ligated) pterygopalatine artery [PTY], (ligated) external carotid artery [ECA], internal carotid artery [ICA] and middle cerebral artery [MCA].

Arteriograms were evaluated without knowledge of the fixation procedure in accordance with a modification of a previously reported topographically rank ordered scale [9]. All arteriograms were evaluated individually and assigned one of the following scores: 0 = patent arteries, 1 = middle cerebral artery occlusion (MCAO in one or more branches), 2 = MCAO at the stem, 3 = distal and intracranial ICA occlusion, 4 = extracranial and proximal ICA occlusion. The angiographic scores are illustrated on Figure 1. During surgery using an unblinded evaluation of the arteriograms, fourteen (39%) of the animals achieved a score of 0, 1 or 1.5 with an occlusion in the distal half of the MCA. These 14 animals were considered poorly embolized and were embolized again by repeating all embolization procedures, and the final grade of occlusion was determined by the repeated arteriography. In two animals embolization and arteriography according to the above procedures were performed 3 times, while the other 12 animals were successfully embolized on the second attempt. In order not to expose animals to an excessive amount of dislocated embolus material, no more than three embolization attempts were allowed in this study. After the final arteriography and then ligations of vessels, femoral and neck wounds were closed. The effect of anaesthesia was reverted with naloxone 0.3 mg/kg s.c. (Narcanti^®^, Du Pont Pharmaceuticals, U.S.A.).

### Clinical Evaluation

About two hours after recovering from anaesthesia, the clinical neurological status was evaluated using a rank ordered scale for rats developed by Bederson et al. [11]; a score of 0 reflected normal neurology and no observable deficit (grade 0), a score of 1 corresponded to moderate clinical damage observed by forelimb flexion (grade 1), a score of 2 reflected severe clinical damage and decreased resistance to lateral push (and forelimb flexion) without circling (grade 2), while a score of 3 corresponded to maximum clinical damage with the same behaviour as grade 2 with circling (grade 3). A Bederson score has been found to correlate to the infarct sizes [11]. Twenty-four hours after anaesthesia all animals were weighted, re-evaluated clinically as above and terminated.

### Preparation of brain tissue and measurement of infarct size

Twelve animals were perfusion fixed with formalin. In this group and in a second group of 13 other animals the brains were carefully removed and immersed in formalin. A third group of 11 animals was left dead for three hours at room temperature before the brains were dissected out and immersed in formalin. The 3 different fixation procedures were evenly distributed without significant differences between the 3 groups with different grades of vascular occlusion. As shown previously, fixation procedures had no influence on the results of this current investigation, because infarct and oedema volumes calculated as percentages of the corresponding hemisphere were independent of fixation procedures [6].

Standard histological procedures were used and coronal serial sections of the brain with a distance of 400 μm between each section (about 20-25 sections per brain) were stained with hematoxylin-eosin [12]. This distance between sections was used, because a tripling of the distance between brain sections can decrease the accuracy of the measurements by up to 4% [13-15].

### Volume calculations using image analysis and planimetry

**Figure 2:**
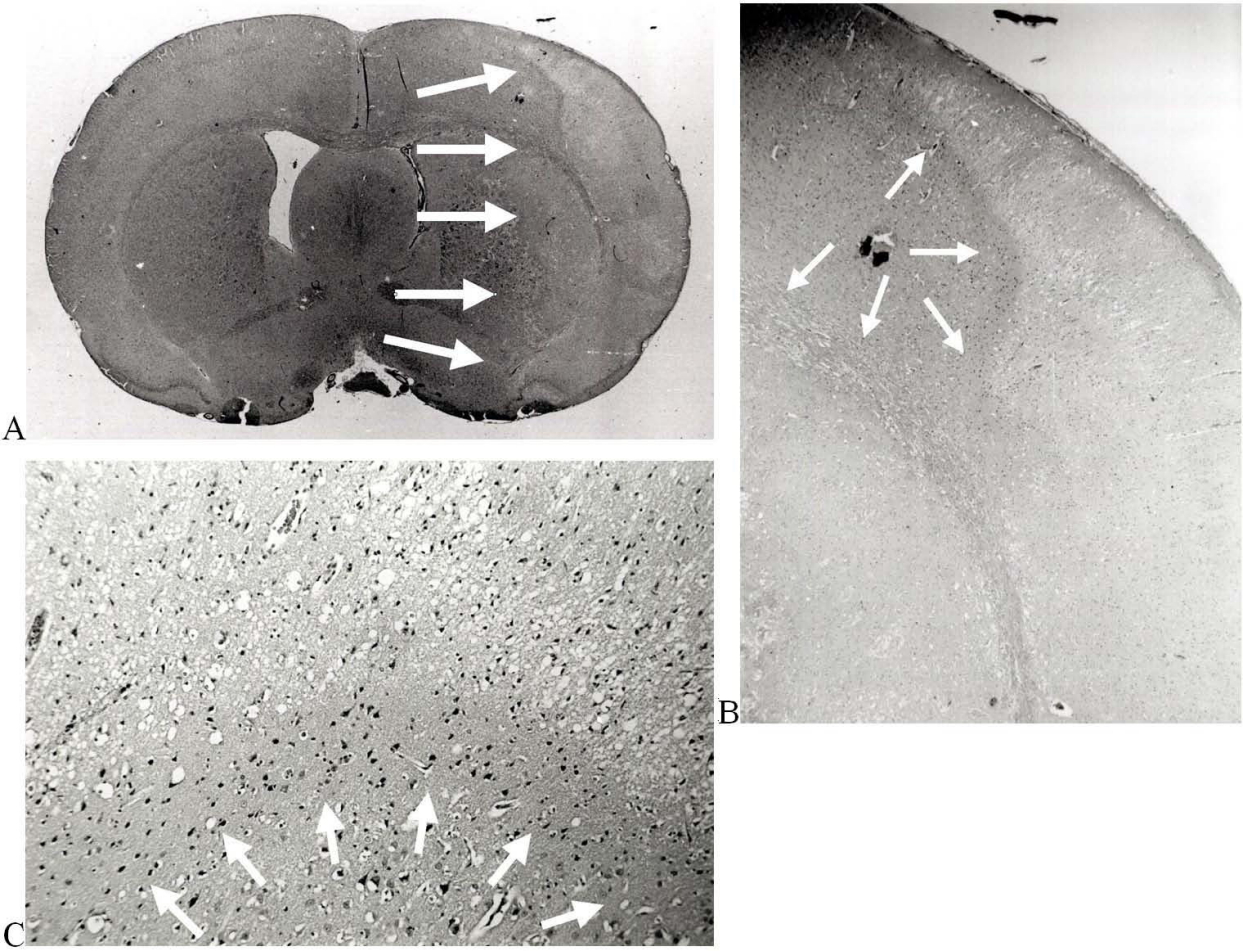
**(a + b + c)**. Hematoxylin-eosin stained paraffin-embedded coronal section of an immersion fixed brain from an embolized rat with a proximal middle cerebral artery occlusion (A). Arrows indicate the border of the infarct, constituting 47% of the volume of the right hemisphere. The borderline of infarction is not easily identified by differences of structure or colour intensity. Medium power magnification (B). The border between healthy and dead brain is more obvious (arrows). High power magnification (C) of the narrow penumbral zone. The border between healthy and live neurons now distinctively exposed (arrows). A rim of 'dark' neurons adjacent to the normal neurons is observed and deeper in the infarct the cell bodies have shrunken from the surrounding neuropil [33,34].

All brain sections were examined carefully by conventional light microscopy by investigators blinded to the experimental protocol. Borderlines of the cortical and subcortical infarcts were delineated and separated from healthy brain regions using a pen, and the infarcted tissue could be recognised easily (Figure 2). All brain sections were scanned into a personal computer by the use of an image analysis program (Sidney Data, Copenhagen, Denmark), providing volume measurements of hemispheres and infarctions. The accuracy of the planimetry was secured by performing repeated control-scans of a rectangle sized 115 mm^2 ^placed in a horizontal as well as in a diagonal position.

### Calculations and analysis of weight, oedema and statistics

Animals were weighted preoperative and immediately before the animal was terminated at 24 hours. Postoperative weight loss in percent of preoperative weight was calculated as: 100 × (preoperative weight - pre-termination weight)/preoperative weight. Under the assumption that differences between right and left hemisphere volumes constituted oedema sizes, the volumes of oedema in percent of the left hemisphere was calculated as: 100 × (RH - LH)/LH, with RH and LH representing volumes of the right and left hemispheres, respectively. Oedema volumes in percent of infarct volumes were calculated as: 10^4 ^× (RH - LH)/(INF × RH), where INF represented infarct sizes in percent of RH. Non-parametric statistical analyses of data were performed, because all data were either categorical, or could be placed on rank ordinal or ratio-interval scales with no apparent normal distribution. Mann-Whitney U-test for unpaired observations, Wilcoxon matched-pairs test for paired observations and the Spearman test for correlation of ranked pairs were used. P-levels below 0.05 were considered significant.

## Results

**Figure 3:**
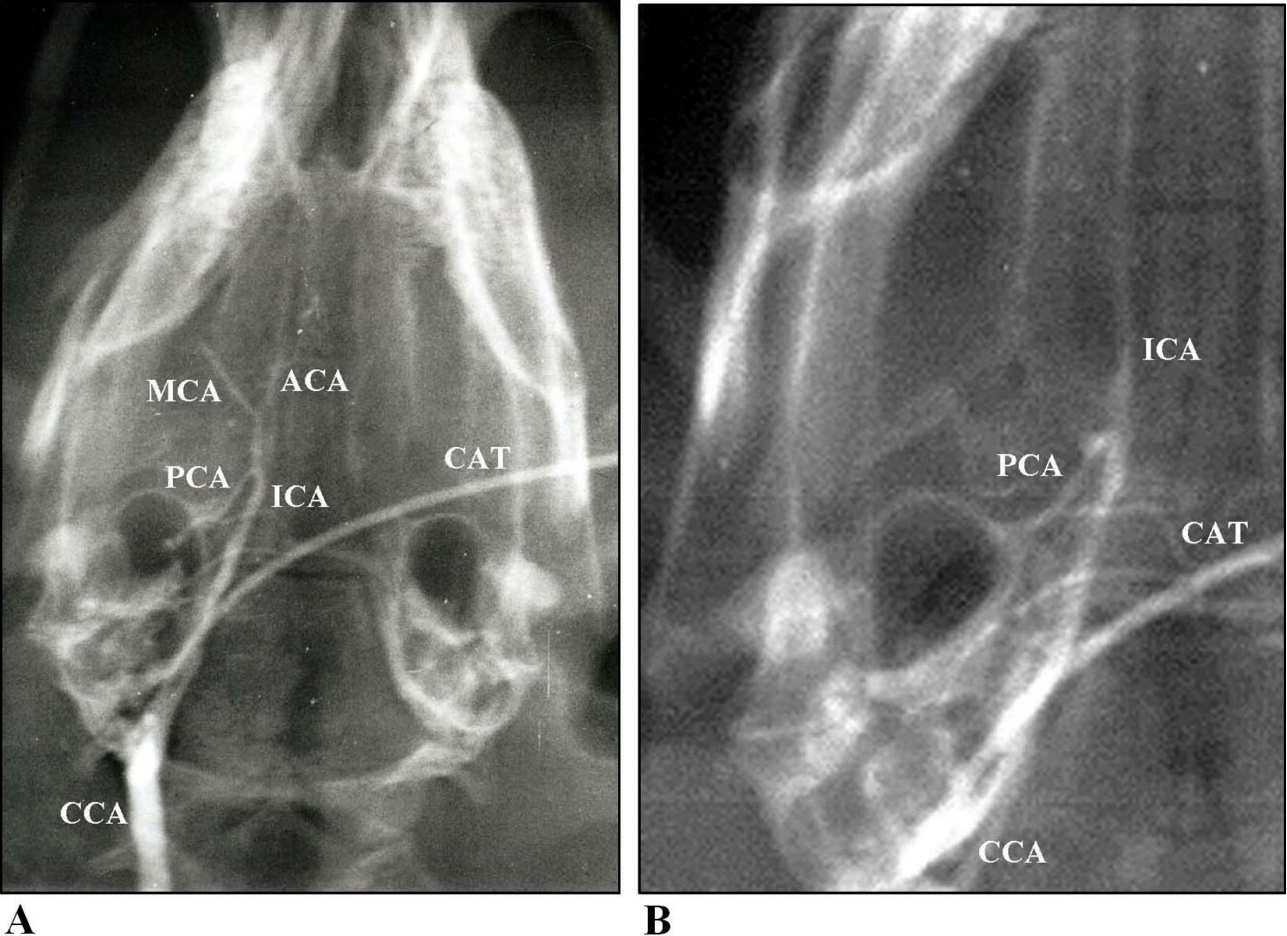
**(a + b)**. Arteriograms showing proximal occlusion of the main-stem of the middle cerebral artery (A) and a distal occlusion of the internal carotid artery (B), arteriographic score values of 2 and 3 respectively. The following arteries are identified: internal carotid artery [ICA], anterior cerebral artery [ACA], middle cerebral artery [MCA], posterior cerebral artery [PCA], common carotid artery [CCA] and catheter [CAT].

On the final blinded evaluation of the arteriograms the surviving animals were divided into 3 different subgroups: one subgroup of 11 animals had a grade 2 (stem of the MCA) occlusion, a second subgroup of 19 animals had a grade 3 (intracranial ICA) occlusion and the remaining subgroup of 5 animals had a grade 4 (extracranial ICA) occlusion. One animal found dead at 20 hours was excluded from the results, and all other animals survived 24 hours. Figures 3A and 3B illustrate grade 2 and 3 embolizations. The 3 different fixation methods were evenly distributed without significant differences between the 3 groups on vascular occlusions.

### Physiological parameters

No significant differences were observed between any of the 3 subgroups in physiological parameters. Results (median and interquartile range) in all 35 animals were: MABP: 76 mmHg (70-85), haemoglobin: 10.4 mmol/l (10.1-10.7), glucose: 11.3 mmol/l (11-13), pH_a_: 7.395 (7.38-7.41), P_a_CO_2_: 39 mmHg (37-42), P_a_O_2_: 87 mmHg (82-92), O_2_-saturation: 96% (96-97), preoperative weight of animals: 350 g (313-397), size of both hemispheres (excluding cerebellum, brainstem and olfactory bulbs): 795 mm^3 ^(740-892), respectively (comparing all subgroups, all p > 0.05, ns). Similar to earlier reports by other investigators [14], we found no influence of rat age or preoperative weight on infarct volume.

### Infarct size was dependent on the efficacy of embolization documented by grade of arteriographic occlusion

As shown in table 1 the 11 animals with a MCAO had median cortical, subcortical and total infarct volumes of: 17.2%, 20.5% and 40% respectively of the right hemisphere volume. The 19 animals with intracranial ICAO had almost identical median infarct volumes of 16.6%, 19.9% and 40% respectively (all p > 0.45). Thus animals with a grade 2 or 3 occlusion obtained similar infarct volumes.

**Table 1:** Main results

Type of occlusion	N	Percent post-operative weight loss	Oedema in percent of the volume of the left hemisphere	Volume of infarct in percent of the right hemisphere		
				Subcortex	Cortex	Total infarct
MCA	11	5.5 (5-6)	5.6 (2-11)	20.5 (9-29)	17.2 (9-18)	40 (23-47)

Intracranial ICA	19	5.9 (5-8)	6.6 (2-12)	19.9 (12-34)	16.6 (13-23)	40 (22-56)

Extracranial ICA	5	4.8* (1-5)	0.8* (0-1)	0.7*** (0-1)	2.0** (1-3)	3.5*** (2-5)

Main results: postoperative weight loss, volumes of oedema and infarction. Median values and values in brackets are 25 and 75 percentiles. The 5 animals with an extracranial ICAO significantly differed from all the other 30 animals with an intracranial site of occlusion. * = p < 0.05, ** = p < 0.01, *** = p < 0.001.

Two of the 5 animals with an extracranial ICAO had in addition an unintended insufficient ligation of the pterygopalatine artery documented by the arteriography. One of the animals with an open pterygopalatine artery had a total infarct of only 1.7% of right hemisphere volume even embolized three times with the first two arteriograms showing patent arteries, the third showing extracranial ICA occlusion. These 5 animals all had significantly lower size of infarcts (table 1); total infarct was in all 5 animals below 7% (median 3.5%) of the right hemisphere volume.

### Repeated embolization improved the occlusion rate

Three animals with MCAO, 8 animals with intracranial ICAO and 3 animals with extracranial ICAO were embolized more than once. In the animals with repeated embolization, the 3 animals with a final extracranial ICAO had a significantly (p < 0.02) lower initial angiographic score of median = 0 = patent arteries compared to all the other 11 repeatedly embolized animals on the arteriogram obtained after the initial embolization. These 14 animals submitted to repeated embolization had a significant higher final than initial arteriographic score value (p < 0.002). Repeating the embolization thus improved successful embolization. When these 14 animals were compared with all other animals embolized only once, the differences in the physiological or other main result parameters were not significant.

### Clinical outcome

At the postoperative clinical score two animals had a score value of 2, all other animals had a score value of 3. Specifically, among animals in the MCA group, immediately after surgery the Bederson score was 3 (3-3), at 24 hours post-surgery the pre-termination score was 1 (0-2) revealing an improvement of 2 (1-3). For the intracranial ICA group the postoperative score was 3 (3-3), the pre-termination score was 2 (1-2) revealing an improvement of 1 (1-2), and in the extracranial ICA group the postoperative score was 3 (3-3), the pre-termination score 0 (0-1) resulting in an improvement of 2 (2-3). The improved pre-termination scores were significant in all groups (P < 0.05), but no group differed from each other when comparing post-surgery or pre-termination scores. Thus generally the neurological deficit remitted significantly as the clinical score decreased from the postoperative level to a median score value of 1 at the observation after 24 hours (all 35 animals; p < 0.0001). The subgroup of 5 animals with extracranial ICAO had a pre-termination median clinical score value of 0 compared to all other animals with a median score value of 1.5 (p = 0.09).

The pre-termination weight of all 35 animals was significantly (p < 0.001) lower than the initial weight of the animals, and the 5 animals with extracranial ICAO had a significantly smaller loss of weight compared to all the other 30 animals (table 1).

### Correlations between infarct size and oedema, clinical outcome and weight loss

Significant correlations were found between cortical, subcortical and total infarct volumes (all animals; all R > 0.76, all p < 0.0001). Other important correlations are listed in table 2.

**Table 2:** Correlations between infarct size and oedema, clinical outcome and weight loss

Correlations: Values of Spearman R	N	Percent post-operative weight loss	Volume of infarct in percent of the right hemisphere		
			Subcortex	Cortex	Total infarct
Oedema in percent of the left hemisphere	35	0.45**	0.82***	0.72***	0.83***

Clinical score pre-termination	35	0.34*	0.51**	0.49*	0.53**

Postoperative weight loss	35		0.55***	0.53***	0.59***

Cortical, subcortical and total infarct volumes each correlated significantly with oedema, clinical score before termination and weight loss. Values in the table are all Spearman R, * = p < 0.05, ** = p < 0.01, *** = p < 0.001.

### The embolized right hemisphere suffered from significant oedema depending on infarct size but not site of occlusion

The embolized right hemisphere was larger than the left in the 30 animals with an intracranial site of arterial occlusion (p < 0.001), except in the 5 animals with extracranial ICAO, where the median size of the hemispheres were 366 mm^3 ^and 363 mm^3^, right and left respectively (p > 0.68). The magnitude of the oedema in each subgroup is presented in table 1. In all 3 subgroups of embolization the size of cerebral oedema ranged from median 11% to 13% of the size of the infarct, with no significant intergroup differences. In the subgroup of 5 animals with an extracranial ICAO the size of cerebral oedema measured in percent of the size of the left hemisphere was significantly smaller (all p < 0.02) in the subgroup of animals with extracranial ICAO compared to either or both subgroups of animals with an intracranial site of occlusion.

### Comparing the present results with results from another experiment

**Figure 4:**
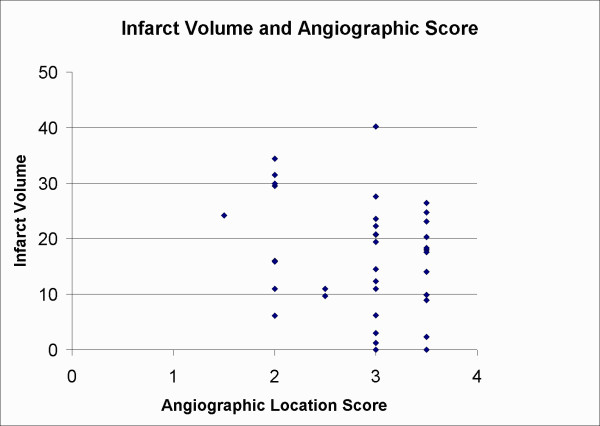
**An illustration of the relationship between infarct volume in percent of a normal hemisphere and angiographic score**. No significant differences were observed, thus different embolization sites resulted in similar infarct volumes (especially grade 2 did not differ from grade 3 or grade 3.5). In this study scoring occlusions between two sites by adding or subtracting a value of 0.5 did angiographic occlusion scores more elaborately. Only animals with occlusion grades from 1.5 (distal MCAO) to 3.5 (ICA occlusion at the origin of the MCA after and proximal to the entrance of the posterior cerebral artery) were included.

We re-examined data from another experiment using a similar animal model, where different stroke treatments had no effect on infarct volumes and where the relationship between infarct volumes and different occlusion sites had not be investigated before [16]. Although the study used smaller emboli of 4 to 6 mm instead of 8 mm [16], occlusion sites were the same and therefore results of emboli locations and the associated infarct volumes should be comparable. In 38 embolized animals [16] we found the results shown in Figure 4. Comparing 8 animals with a grade 2 occlusion with 14 animals with a grade 3 occlusion or 13 animals with a grade 3.5 occlusion we found no significant differences in infarct volumes (p = 0.23 and 0.25 respectively). Figure 1 illustrates different angiographic scores. Similarly a grade 3 occlusion did not differ from a grade 3.5 occlusion (p = 0.96). Occlusions in the right hemisphere of the ICA between the posterior cerebral artery (PCA) and the MCA was scored as 3 if the occlusions were in the upper half more proximal to the MCA, or 3.5 when occlusions happened in the lower half more proximal to the PCA (these types of occlusions would both block the entrance to the right MCA). Thus these results indicate that occlusions scoring from 1.5 to 3.5 provide similar infarct volumes. These independent results confirm and extend earlier findings in this study, where grade 2 or 3 occlusions resulted in similar infarct volumes.

## Discussion

The present study using a 'arterial-like' single macroclot model [10] demonstrated a significant relation between the sites of angiographically verified embolization and histologically verified cerebral infarction and oedema when comparing extracranial ICA occlusions to intracranial MCA or ICA occlusions. Consequently, the position of the arterial occlusion significantly influenced the size of the infarct and oedema in this rat embolic stroke model. The relation found between the location of the vascular occlusion, size of the infarct, oedema formation, clinical score and postoperative weight loss demonstrates the similarities to human stroke and the robustness of this model.

The lack of infarct volume differences when comparing animals with a grade 2 or 3 occlusion may seem contrary to clinical findings, where distal ICA occlusions have been associated with a worsening of outcome compared to MCA stem occlusions [17-19]. In human stroke the worsening of outcomes by ICA occlusions may be explained by larger emboli unable to propagate by the MCA and causing profound disturbances of blood flow in the circle of Willis. The size of emboli was similar for all animals in this experimental stroke model, but a possible fragmentation or folding of an embolus may affect its shape and explain the variance in embolus locations. Furthermore outcome in human stroke is generally measured by reperfusion or behavioural scores [17-19], which may or may not correlate well with infarct volumes. In humans with larger MCA infarcts, the ICA was occluded in 40% of the patients [20]. If the collateral blood supply was sufficient as in an extracranial ICA occlusion, humans may show no symptoms of an ICA occlusion, similar to our present observations in rats. If an ICA occlusion had major impact upon collateral blood supply, corresponding to an embolus or thrombus extending into the circle of Willis, large infarctions have been shown in humans [20]. These findings are similar to our present results of grade 3 or 3.5 occlusions in rats, where the ICA occlusion extended into the circle of Willis and blocked any collateral blood supply from entering the MCA.

In another study of 55 humans with complete MCA infarctions or more extended infarcts mortality was 78% [21], exemplifying the need for models with high translational value in order to facilitate treatment investigations of this severe disease.

In humans bad clinical outcome has been predicted by the presence of hemianopia and reduced consciousness, where especially coma predicted death [20]. Such symptoms of hemianopia may be difficult to measure after embolization in rats, but reduced consciousness or coma may respond well to a Bederson score of 2 or 3, where an animal becomes less responsive and shows clear signs of neurological damage. The Bederson Grading System has been linked to infarct sizes (a Bederson score of 3 has been linked to larger infarcts and worse outcome than lower scores).

In older investigations where we used our multiple 'arterial-like' microclot method [5,9] the mortality immediately after embolization was up to 20% caused by respiratory arrest, probably induced by massive embolization including the brain stem injuring the respiratory centre. In addition the infarcts were larger; 40-60% of the hemisphere volume, resulting in a further 30-90% mortality within the first 4-6 days following embolization, which correlated to the volume of suspension of emboli and the size of the infarct. This large technically induced mortality was a drawback of the multiple 'arterial-like' microclot method, but fortunately was not the result of the present model, where we used only one embolus per occlusion. Using the present single-clot model, and by comparing animals receiving one embolus with animals needing more than one embolus to obtain an occlusion, animals did not differ in infarct sizes or mortality. In theory, by the re-embolization of animals failing to show an occlusion after receiving the first embolus a bias may be introduced, but human stroke patients often experiences multiple infarcts and emboli [22-24]. Thus, using multiple embolizations was not a drawback of the present animal model, but may strengthen its translational capacities.

Theoretically we did not have to consider occlusions in the distal half of the MCA, corresponding to animals with less than a grade 2 occlusion, as poor, because grade 1 or 1.5 occlusions may generate a similar amount of brain damage as grade 2 or 3 occlusions. This trend is shown in Figure 4, where a grade 1.5 occlusion provided large brain damage. In practice it was difficult to verify occlusions of the distal half of the MCA, as the diameter of the MCA becomes gradually smaller and the MCA turns and continues out of the plane of the angiography. Radiation diffractions and reflections made it difficult to see the small and distal parts of the MCA clearly, and often we found that seemingly valid occlusions of the distal half of the MCA showed no evidence of occlusion by later examinations. Therefore we considered embolizations of less than grade 2 to be unreliable and chose to re-embolize those animals. If future investigations are able to measure occlusions of the distal half of the MCA reliably, such results may be of great translational interest to humans. Recently investigators found that M1 MCA occlusions in humans, corresponding to grade 1 to 2.5 occlusions in the rat, were independent risk factors of poor outcome, compared to M2 occlusions, corresponding to occlusion grades of less than 1 in the rat [25]. Thus both in rats and humans occlusions of the proximal part of the MCA have been linked to the worst outcomes.

Though this experimental stroke model has been designed to mimic human stroke elaborately differences related to variation of emboli sizes and associated collateral blood supply may pose restrictions upon the similarity, which further strengthens the relevance of the present study as an evaluation of the translational capacities of this embolic stroke model. We have been unable to find a relation between infarct size and emboli location, comparing similar sizes of emboli occluding the stem of MCA or the distal part of ICA, in human stroke. This current animal model was the first to demonstrate that the time-window of thrombolysis in rats was identical to the time-window of thrombolysis in human ischaemic stroke reported a couple of years later (compare references [3] and [26]). Thus, should future investigations find that this rat model of embolic stroke differs from human stroke in outcome and infarct sizes, even if similar emboli locations are observed, there is still ample evidence to suggest a high degree of translational value of this animal model. Also, in studies of treatment effect after embolic stroke, documentation of evenly distributed degree of ischemia (e.g. by angiography) among treatment and control groups, before treatment is initiated, is favorable in order to secure a similar amount of brain damage among animals in each group, especially when groups are small in numbers. Our current study shows that an evenly distributed degree of ischemia is obtained from grade 2 (main stem MCA) and grade 3 (distal ICA) occlusions (even grades 2, 3 and 3.5 were found to result in similar infarctions). This important finding illustrates that investigators do not need to obtain a similar number of animals with a grade 2 or 3 in each experimental group as no significant differences when comparing subcortical, cortical or total infarct volumes were found, while animals with grade 4 occlusions should be evenly distributed or excluded.

In rats abundant collaterals exist between distal branches of the anterior cerebral artery, MCA and the posterior cerebral artery [27]. A proximal extracranial ICAO increases the chance of collateral circulation also by the circle of Willis. In the rat, the pterygopalatine artery branches off from the ICA, ligation of the artery is therefore necessary to achieve embolization only of the ICA and MCA. The type of vascular occlusion might influence the extent of infarction.

Determining infarct size at 24 hours as done in this study carries the risk of overestimating the volume caused by oedema of the infarcted tissue. However, at day 7 the process of resorption [12] results in ~30% shrinkage of a large embolic infarct [28]. We therefore chose 24 hours as an early time point where the infarct was fully recognised, before severe swelling or shrinkage occurs.

The magnitude of oedema depends of the choice of ischaemic model and the timing [12]. Animals can die from herniation caused by large hemispheric infarcts and ipsilateral brain oedema within 24-48 hours [29]. As shown previously the three different fixation procedures did not interfere with the measurement of oedema, which after 24 hours constituted 11% of the infarct volume [6], compared to 6% using comparable methods [28], provided all enlargement of the right hemisphere was located inside the infarction, which may not be absolutely valid [30]. The calculations reported by Leach et al. [31] and Swanson et al. [32] compensate for these problems, and the volume effect of oedema is attempted weighted out. The grade of oedema was correlated to the clinical outcome and infarct volume, and the size of the right and the left hemisphere correlated significantly to each other, but not to the rat age or preoperative weight.

In conclusion, extracranial ICA occlusions (grade 4) provided significant less infarction than occlusions of the intracranial and distal part of ICA (grade 3) or the stem of the MCA (grade 2). No differences were found when comparing infarctions resulting from grade 2 or 3 occlusions. This observation was further strengthened by the re-examination of data from another study based upon a similar model. Thus when groups are small in numbers, animals with grade 4 occlusions should be evenly distributed among groups, while grade 2 and 3 occlusions may be intermixed at random. This observation was further strengthened as similar significant correlations were found between a) cortical, subcortical and total infarct volumes and oedema in percent of the left hemisphere, b) clinical score before termination and c) postoperative weight loss among animals with grade 2 or 3 occlusions.

The lack of differences between grade 2 and 3 occlusions simplify the surgical procedures, thereby making the use of this embolic stroke model or other MCAO models a valuable asset for translational stroke research.

## Competing interests

The authors declare that they have no competing interests.

## Authors' contributions

All authors have made substantive intellectual contributions to this study, including conception and design, acquisition of data, analysis and interpretation of data, writing and revising the manuscript. All authors have given final approval of this current version. Each author has participated sufficiently in acquisition of funding and the collection of data.
